# Complex Multilevel Control of Hemolysin Production by Uropathogenic Escherichia coli

**DOI:** 10.1128/mBio.02248-19

**Published:** 2019-10-01

**Authors:** Nguyen Thi Khanh Nhu, Minh-Duy Phan, Brian M. Forde, Ambika M. V. Murthy, Kate M. Peters, Christopher J. Day, Jessica Poole, Timothy J. Kidd, Rodney A. Welch, Michael P. Jennings, Glen C. Ulett, Matthew J. Sweet, Scott A. Beatson, Mark A. Schembri

**Affiliations:** a School of Chemistry and Molecular Biosciences, The University of Queensland, Brisbane, Queensland, Australia; b Australian Infectious Diseases Research Centre, The University of Queensland, Brisbane, Queensland, Australia; c Australian Centre for Ecogenomics, The University of Queensland, Brisbane, Queensland, Australia; d Institute for Molecular Bioscience (IMB) and IMB Centre for Inflammation and Disease Research, The University of Queensland, Brisbane, Queensland, Australia; e Institute for Glycomics, Griffith University Gold Coast Campus, Gold Coast, Queensland, Australia; f Department of Medical Microbiology and Immunology, School of Medicine and Public Health, University of Wisconsin, Madison, Wisconsin, USA; g School of Medical Sciences, and Menzies Health Institute Queensland, Griffith University, Southport, Australia; Washington University School of Medicine; University of Michigan; National University of Singapore and Genome Institute of Singapore

**Keywords:** *Escherichia coli*, TraDIS, gene regulation, hemolysin, urinary tract infection, virulence

## Abstract

Uropathogenic Escherichia coli (UPEC) is the major cause of urinary tract infections. Nearly half of all UPEC strains secrete hemolysin, a cytotoxic pore-forming toxin. Here, we show that the prevalence of the hemolysin toxin gene (*hlyA*) is highly variable among the most common 83 E. coli sequence types (STs) represented on the EnteroBase genome database. To explore this diversity in the context of a defined monophyletic lineage, we contextualized sequence variation of the *hlyCABD* operon within the genealogy of the globally disseminated multidrug-resistant ST131 clone. We show that sequence changes in *hlyCABD* and its newly defined 1.616-kb-long leader sequence correspond to phylogenetic designation, and that ST131 strains with the strongest hemolytic activity belong to the most extensive multidrug-resistant sublineage (clade C2). To define the set of genes involved in hemolysin production, the clade C2 strain S65EC was completely sequenced and subjected to a genome-wide screen by combining saturated transposon mutagenesis and transposon-directed insertion site sequencing with the capacity to lyse red blood cells. Using this approach, and subsequent targeted mutagenesis and complementation, 13 genes were confirmed to be specifically required for production of active hemolysin. New hemolysin-controlling elements included discrete sets of genes involved in lipopolysaccharide (LPS) inner core biosynthesis (*waaC*, *waaF*, *waaG*, and *rfaE*) and cytoplasmic chaperone activity (*dnaK* and *dnaJ*), and we show these are required for hemolysin secretion. Overall, this work provides a unique description of hemolysin sequence diversity in a single clonal lineage and describes a complex multilevel system of regulatory control for this important toxin.

## Abstract (precis)

Uropathogenic E. coli (UPEC) is the major cause of urinary tract infections and a frequent cause of sepsis. Nearly half of all UPEC strains produce the potent cytotoxin hemolysin, and its expression is associated with enhanced virulence. In this study, we explored hemolysin variation within the globally dominant UPEC ST131 clone, finding that strains from the ST131 sublineage with the greatest multidrug resistance also possess the strongest hemolytic activity. We also employed an innovative forward genetic screen to define the set of genes required for hemolysin production. Using this approach, and subsequent targeted mutagenesis and complementation, we identified new hemolysin-controlling elements involved in LPS inner core biosynthesis and cytoplasmic chaperone activity, and we show that mechanistically they are required for hemolysin secretion. These original discoveries substantially enhance our understanding of hemolysin regulation, secretion and function.

## INTRODUCTION

Uropathogenic Escherichia coli (UPEC) is the primary cause of urinary tract infection (UTI) and a frequent cause of sepsis, diseases of major significance to global human health and increasingly associated with antibiotic resistance (1–3). Many UPEC strains belong to globally disseminated clones that can be differentiated based on their multilocus sequence type (ST), including ST69, ST73, ST95, and ST131 (4–6). Among these, ST131 represents the predominant fluoroquinolone-resistant clone worldwide and the most frequent cause of UTI and urosepsis (7–11). Despite the presence of unique features that define ST131 and other clones, strains within these phylogenetically related lineages also exhibit extensive diversity in their accessory genome. This occurs primarily through the possession of multiple large genomic islands that contain different combinations of genes encoding virulence factors such as adhesins (e.g., fimbriae and autotransporters), surface polysaccharides (e.g., capsule and O antigen), iron acquisition systems (e.g., siderophores and heme scavenging systems), and toxins (e.g., hemolysin and cytotoxic necrotizing factor-1) that are associated with the capacity to cause disease (10, 12, 13).

Hemolysin, a prototype member of the repeats-in-toxin (RTX) family, is a potent pore-forming toxin secreted by 40 to 50% of all UPEC strains (14). The production of hemolysin is strongly associated with UPEC strains that cause pyelonephritis and urosepsis, suggesting a link with increased virulence (14–18). The genes responsible for the production, maturation, and secretion of hemolysin include the genomic island (GI)-located *hlyCABD* operon, and the distally located *tolC* gene (19, 20). HlyA is translated intracellularly as a nontoxic prohemolysin (proHlyA), which is then acylated at Lys564 and Lys690 by the HlyC acyltransferase (21–23). The active HlyA toxin is exported through a type I secretion system (T1SS) that contains an ATP-binding cassette transporter, HlyB, the membrane fusion protein HlyD, and the outer membrane TolC protein (20, 24). Correct folding and stabilization of HlyA also require the cofactor Ca^2+^, which binds to glycine-rich repeats in the toxin (25, 26).

UPEC strains that possess either strong or weak hemolytic activity have been described, as well as different effects of hemolysin on host cells at lytic and sublytic doses (27–32). While these observations have been linked to differential expression of hemolysin (32–34), the precise genetic basis for such differences remains to be properly elucidated. Hemolysin expression is regulated by several environmental stimuli, including temperature, oxygen, and osmolarity (35, 36). The histone-like nucleoid structuring protein H-NS, an important global regulator that controls the transcription of multiple genes associated with UPEC virulence, represses transcription of the *hlyCABD* genes (37, 38). Another regulator that senses environmental stimuli and responds to stress, CpxR, negatively regulates hemolysin production (31). The noncoding region upstream of the *hlyCABD* coding sequences also plays an important role in hemolysin regulation (34, 39–41). This region contains an 8-bp sequence termed the operon polarity suppressor (*ops* [GGCGGTAG]) element, where RfaH, a transcriptional antiterminator, binds and controls the transcription of the *hlyCABD* genes (42–46). Other features of this noncoding region, including characterization of the promoter element, remain poorly defined. In the context of UTI, the function of hemolysin has been associated with exfoliation of uroepithelial cells in mice (47) and human bladder organoids (48), as well as inhibition of the proinflammatory cytokine interleukin-6 (IL-6) from human bladder epithelial cells (49) and peritoneal macrophages (50). We recently showed that hemolysin activates the NOD-like receptor pyrin domain-containing 3 (NLRP3) inflammasome and triggers macrophage cell death (30, 32, 51). We also found that variation in hemolysin expression by UPEC can have profound effects on biological outcomes; low-level hemolysin expression triggers NLRP3-mediated macrophage cell death that is associated with host protection in a mouse model of experimental UTI, whereas high-level hemolysin expression triggers NLRP3-independent macrophage cell death and increased bladder colonization (32).

Despite the above knowledge, a complete understanding of the molecular mechanisms that control hemolysin production remains to be fully elucidated. In addition, the genetic basis for variation in the level of hemolysin expression between different UPEC strains has not been resolved. In this study, we investigated the prevalence of hemolysin genes in the context of major UPEC clones and then used our in-depth knowledge of the genealogy of ST131 to assess variation within a single lineage. Analysis of the *hlyCABD* operon indicates that variation in hemolysin expression between different ST131 strains is primarily related to sequence differences in the very long 5′-untranslated leader sequence and *hlyCABD* coding regions, and these variations follow a clade-specific association. We also describe the application of an innovative genome-wide high-throughput forward genetic screen to identify the set of genes involved in the production of active hemolysin, measured by the capacity to lyse red blood cells. This unique approach revealed a requirement for lipopolysaccharide (LPS) inner core biosynthesis and cytoplasmic chaperones for UPEC hemolytic activity, providing major conceptual advances in our understanding of how the secretion of this important toxin is controlled.

## RESULTS

### Distribution of the *hlyA* gene varies among major UPEC clones.

**FIG 1:**
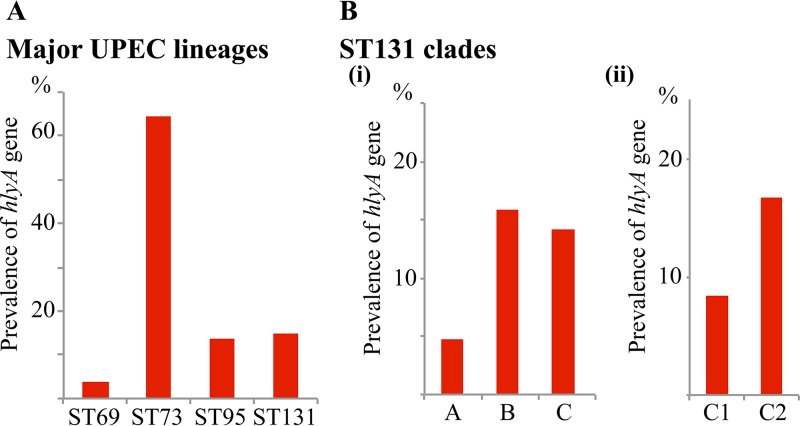
Prevalence of the *hlyA* gene. (A) Prevalence of *hlyA* in strains from the ST69, ST73, ST95, and ST131 UPEC lineages retrieved from EnteroBase. The percentage of strains containing *hlyA* was determined by BLASTn against *hlyA*^CFT073^, with the cutoff at 95% nucleotide identity. (B) Prevalence of *hlyA* in different ST131 clades (i) and in subclades C1 and C2 (ii). The percentage of *hlyA* was determined as described above. ST131 strains were categorized based on clade-specific SNPs as defined in reference 10. Based on this typing, 184 isolates (5.4%) could not be allocated into any of the clades and were therefore excluded from this analysis. We note that 73/184 of these strains contained the *hlyA* gene.

The production of hemolysin is most frequently associated with UPEC strains that cause severe UTI (52–55). To investigate the prevalence of hemolysin genes in the most common E. coli STs, we used E. coli genomes from EnteroBase, a large publicly available *Enterobacteriaceae* genome sequence database (56). Analysis of randomly selected genome assemblies from the 83 highest represented STs revealed a perfect linear correlation between the presence of *hlyA* and *hlyCABD*, demonstrating that the *hlyA* gene is always located within the *hlyCABD* operon (see Fig. S1A in the supplemental material). Further examination of these 83 STs showed extensive variation in the distribution of the *hlyA* gene, with the highest prevalence in ST12, -73, and -127 (79 to 89%; all phylogroup B2), midrange prevalence in ST38, -59, -131, -141, -372, -405 (12 to 49%; phylogroup B2/D), and ST29 (26%; phylogroup B1), and low prevalence (≤9%) in the remaining STs (Fig. S1B). The four most dominant pandemic UPEC lineages, which are highly represented in EnteroBase, were also investigated as a complete data set (downloaded in July 2018). In concordance with the data from 83 STs, the prevalence of *hlyA* in these lineages was significantly higher in ST73 (605/936; 64.6%) compared to ST131 (502/3,391; 14.8%), ST95 (116/859; 13.5%), and ST69 (26/684; 3.8%) (*P* < 0.0001, Chi-square test) (Fig. 1A).

10.1128/mBio.02248-19.2 FIG S1 Prevalence of *hlyA* and *hlyCABD* from the top 83 E. coli sequence types (STs). One hundred strains were randomly chosen from each E. coli ST from EnteroBase. The presence of *hlyA* and *hlyCABD* genes were determined in these assemblies using BLASTn and the corresponding gene(s) retrieved from the complete genome of CFT073 (accession no. AE014075.1) as a search tool, with a cutoff at 90% nucleotide identity and 80% length coverage. (A) Pearson’s correlation indicates perfect linear correlation between the presence of *hlyA* and *hlyCABD*, suggesting the *hlyA* is mostly carried within the whole *hly* operon. (B) Prevalence of *hlyA* in each E. coli ST. (ST without *hlyA* is not displayed.) Download FIG S1, TIF file, 1.9 MB. Copyright © 2019 Nhu et al. 2019 Nhu et al. This content is distributed under the terms of the Creative Commons Attribution 4.0 International license.

Despite the enormous diversity of UPEC at the genome level, the ST131 lineage represents a monophyletic clone, and its genealogy has been well characterized (4, 9, 10, 57, 58). The ST131 clone is comprised of three major sublineages: clades A and B and the fluoroquinolone-resistant clade C. Based on clade-defining single-nucleotide polymorphisms (SNPs) characterized previously (10), 94.6% (3,207/3,391) of the ST131 strains from EnteroBase were classified into their specific clade designation with the following distribution: clade A (*n* = 300), clade B (*n* = 70) and clade C (*n* = 2,737). The *hlyA* gene was found more frequently in strains from clade B (15.9%) and clade C (14.1%), compared to clade A (4.7%) (Fig. 1B). Within clade C, the presence of *hlyA* was significantly more common in strains from the multidrug-resistant subclade C2 (16.7%) compared to subclade C1 (8.4%) (*P* < 0.0001; Chi-square test) (Fig. 1B).

### Variability in hemolysin expression in ST131 correlates with strain phylogeny.

**FIG 2:**
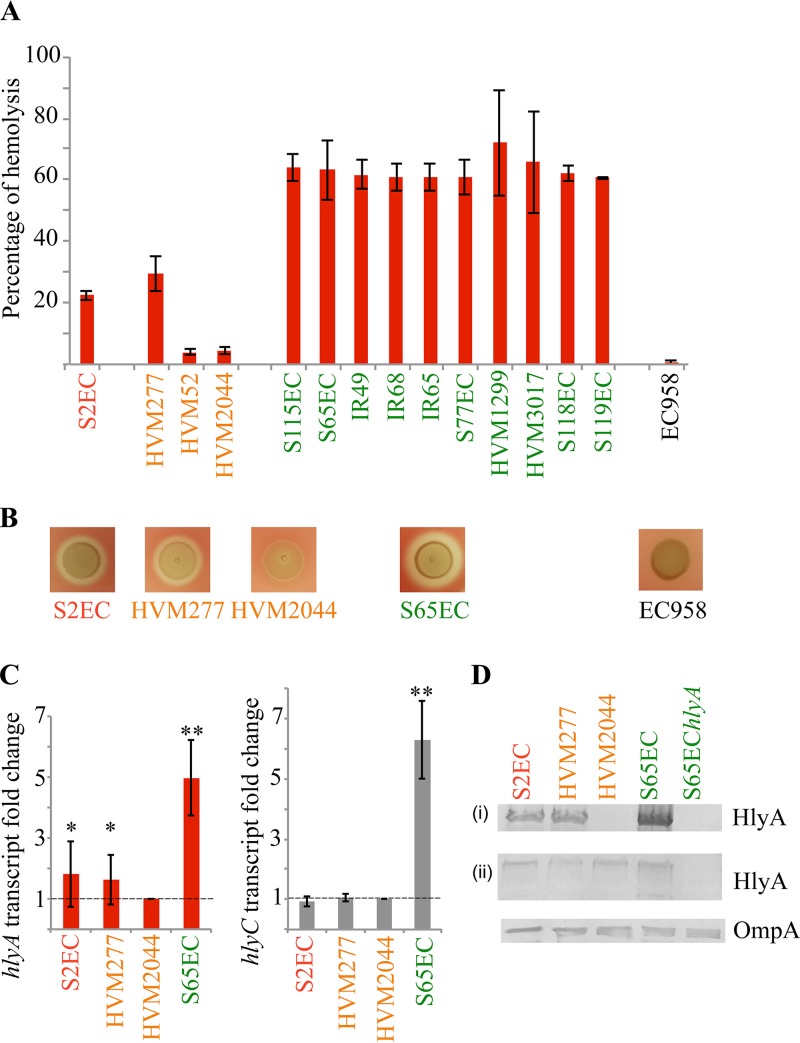
Hemolytic activity of ST131 strains. (A) Percentage of red blood cell hemolysis observed in liquid suspension assay. Strain names are color coded according to their clade designation; red, clade A; orange, clade B; green, clade C. Error bars denote the standard deviation of three biological replicates. (B) Zone of red blood cell hemolysis observed on blood agar. Hemolysin-positive strains are indicated and color coded according to their clade designation; red, clade A; orange, clade B; green, clade C. EC958, which does not contain the *hlyCABD* genes, was used as a negative control. Data are representative of three independent experiments. (C) Comparison of *hlyA* and *hlyC* transcription levels in representative ST131 strains. Relative fold change in the transcription level of *hlyA* and *hlyC* in S2EC (clade A), HVM277 (clade B), and S65EC (clade C) compared to HVM2044 (which expresses the lowest level of hemolysin) was assessed via qRT-PCR, with *gapA* as an endogenous control. Results are displayed as the mean fold change with standard deviation of three biological replicates. The horizontal dashed line represents a fold change of 1, indicating no difference in the transcription level compared to HVM2044. Asterisks denote statistically significant differences as follows: *, *P* < 0.05; **, *P* < 0.0001. (D) Western blot analysis with HlyA-specific antibody in representative ST131 strains, performed using both concentrated supernatant (i) and whole-cell lysates (ii), with OmpA-specific antibody as the loading control.

Hemolysin was originally described as a factor that promoted enhanced virulence in an experimental rat model of peritonitis (59), with subsequent studies revealing a complicated picture of variable hemolysin expression in different unrelated hemolysin-positive UPEC strains (34, 40, 41, 60, 61). We hypothesized new insight into hemolysin biology could be gained by studying this variation in the context of a defined phylogenetic lineage and thus investigated the level of hemolysin expression among *hlyCABD*-positive strains in our previously published ST131 collection (9). The *hlyA* gene was found in 14/95 (14.7%) of strains with the following distribution: clade A = 1 strain, clade B = 3 strains, and clade C = 10 strains (all of which belonged to subclade C2). Hemolysin expression was quantified based on the level of red blood cell hemolysis, revealing that the clade C strains were all strongly hemolytic (∼63% hemolysis [Fig. 2A]). In contrast, the clade B strains (range, 4 to 29% hemolysis) and clade A strain (∼22% hemolysis) were less hemolytic (Fig. 2A). These levels were congruent with analyses based on the size of the zone of hemolysis on blood agar, which also showed that the ST131 clade C strains were the most hemolytic (Fig. 2B).

We also investigated the impact of hemolysin expression on virulence by examining the ability of representative strains to kill human macrophages. Comparative analysis of the strongly hemolytic clade C strain S65EC and the weakly hemolytic strains S2EC (clade A) and HVM277 (clade B) revealed a similar pattern with respect to macrophage cell death; i.e., using a multiplicity of infection equal to 10, S65EC caused ∼60% cell death at 8 h postinfection compared to ∼30% cell death caused by S2EC and HVM277 (see Fig. S2 in the supplemental material).

10.1128/mBio.02248-19.3 FIG S2 Hemolysin expression level affects the ability to trigger cell death of human macrophages. HMDMs were infected with ST131 strains expressing different levels of hemolysin with a range of multiplicities of infection (MOI). Cell death was measured at 2, 8, and 24 h postinfection by lactate dehydrogenase (LDH) release assay. Data (mean + standard error of the mean [SEM]) are combined from four independent experiments using a different donor and are color coded according to the ST131 clade designation of the corresponding strain (red, S2 from clade A; orange, HVM277 from clade B; green, S65EC from clade C). Asterisks indicate statistical significance determined using two-way ANOVA (*, *P* < 0.05; **, *P* < 0.001). Download FIG S2, TIF file, 1.7 MB. Copyright © 2019 Nhu et al. 2019 Nhu et al. This content is distributed under the terms of the Creative Commons Attribution 4.0 International license.

### Hemolysin gene transcription and hemolysin expression correlate with the level of hemolytic activity.

To explore the basis of differential hemolytic activity in ST131, we compared the *hlyA* and *hlyC* transcript levels from selected clade A (S2EC), clade B (HVM277), and clade C (S65EC) strains against HVM2044, the least hemolytic clade B strain (Fig. 2B). Analysis of *hlyA* transcription by qRT-PCR revealed significantly higher transcript levels in S65EC (∼5-fold increase), S2EC (∼1.8-fold increase) and HVM277 (∼1.6-fold increase) compared to HVM2044 (Fig. 2C). Similarly, *hlyC* transcript levels were high in S65EC (∼6.3-fold increase compared to HVM2044), but low in S2EC and HVM277 (levels virtually identical to HVM2044) (Fig. 2C). The level of secreted hemolysin corresponded with these transcript levels, with strongest expression observed in S65EC, the most hemolytic strain (Fig. 2D). Taken together, these data showed for the first time that the variation in hemolytic phenotype between strains from different ST131 clades occurs due to differences in transcription of the *hlyCABD* genes.

### Sequence polymorphisms in the *hlyCABD* untranslated leader transcript correspond with differential hemolysin gene transcription.

**FIG 3:**
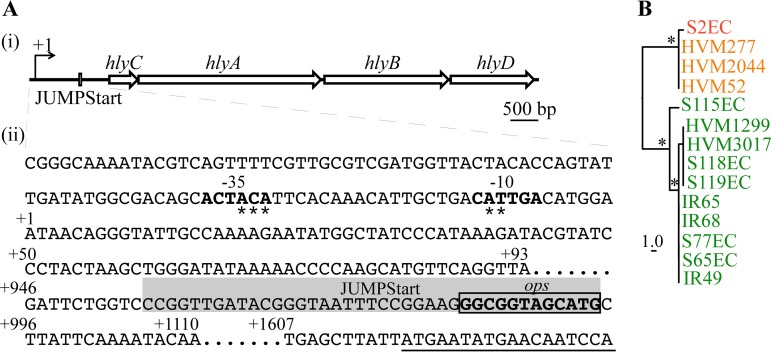
Transcriptional start site of *hlyC*. (A) Diagram (i) and the sequence (ii) of *hlyCABD* operon and its upstream regions. The predicted −10 and −35 regions are bold, with asterisks denoting nucleotides identical to the corresponding E. coli consensus sequences (−10, TATAAT; −35, TTGACA). The *ops* sequence (gray shaded box) is indicated within the JUMPStart sequence (gray shaded), and the *hlyC* coding sequence is underlined. (B) Phylogenetic analyses of the *hlyC* 1.616-kb upstream region, with 1,000 bootstraps. Strain names are color coded according to their clade designation: red, clade A; orange, clade B; green, clade C. The scale indicates the number of substitution SNPs. Asterisks denote branches with a supported bootstrap value of >90%.

Although it has been shown that variation in the region upstream of the *hlyCABD* coding sequence affects hemolysin expression (34, 40, 61), identification of the promoter element of this chromosomal locus has remained elusive. We mapped the transcriptional start site of the *hlyCABD* operon in S65EC using 5′-rapid amplification of cDNA ends (5′-RACE) to a distant 1,616 nucleotides upstream from the *hlyC* start codon (Fig. 3A; see Fig. S3 in the supplemental material). This very long leader transcript contains an *ops* element located 634 bp upstream of the *hlyC* start codon and within a putative 39-bp JUMPStart sequence (Fig. 3A), a common element found in the regulatory region of RfaH-activated genes (62). Comparison of this 1,616-kb leader sequence in our *hlyCABD*-positive ST131 strains revealed phylogenetic clustering into two well-supported groups that matched the hemolysin expression profile of our strains: one for the region from clade A and B strains, and the other for clade C strains, with 16 to 18 SNPs separating the two groups (Fig. 3B). No sequence differences were detected within the JUMPStart element. The promoter element associated with this transcription start site was conserved in all strains examined and contains degenerate −10 and −35 regions (Fig. 3A).

10.1128/mBio.02248-19.4 FIG S3 Electropherogram of the dCTP tailing at the 3′end of the cDNA of *hlyC* by 5′ RACE. Electropherogram of the dCTP tailing at the 3′ end of the cDNA of *hlyC* by 5′ RACE shows the transcription start site of *hlyC* (denoted by the bent arrow). Briefly, *hlyC* mRNA was converted to cDNA using *hlyC*-specific primer hlyC_GSP1. A homopolymeric tail was then added to this cDNA using terminal deoxynucleotidyl transferase (TdT) and dCTP. This product was then sequenced using hlyC_GSP14. Download FIG S3, TIF file, 0.7 MB. Copyright © 2019 Nhu et al. 2019 Nhu et al. This content is distributed under the terms of the Creative Commons Attribution 4.0 International license.

### Hemolysin gene sequences correspond to strain clade designation.

**FIG 4:**
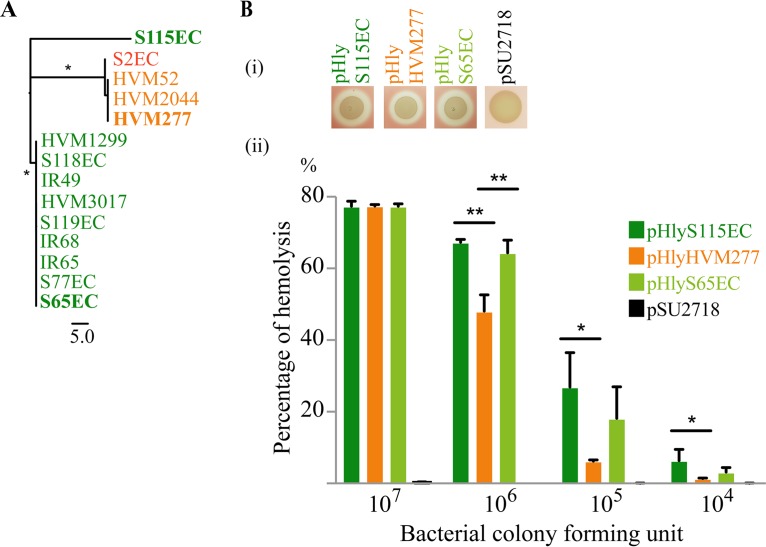
Sequence variation in HlyA. (A) Phylogenetic analysis of the *hlyA* gene retrieved from ST131 assemblies (10), with 1,000 bootstraps. Strain names are color coded according to their clade designation: red, clade A; orange, clade B; green, clade C. The scale indicates the number of substitution SNPs and asterisks denote branches with supported bootstrap values >90%. (B) Overexpression of three sequence variants of hemolysin. All three recombinant constructs were hemolytic on sheep blood agar (i) and in broth (ii). (ii) Overnight cultures of MG1655 containing different hemolysin variants were 10-fold serially diluted, and incubated with LB + 5% sheep blood cells for 3 h at 37°C. At a high concentration (i.e., 10^7^ CFU), all three HlyA variants possessed equivalent hemolytic activity. At a lower concentration, the HlyA^HVM277^ variant (pHly^HVM277^) was the least hemolytic. Results are displayed as the mean and standard error of the mean from three biological replicates. Asterisks represent statistically significant difference: *, *P* < 0.05; **, *P* < 0.0001.

We also examined the level of sequence variation for individual genes in the *hlyCABD* operon. Sequence analysis showed that the *hlyA* gene divided into two well-supported groups, separating clade C strains from clade A/B strains with 28 to 29 SNPs (Fig. 4A). The exception was the outlier strain S115EC (clade C), which contained 37 SNPs in *hlyA* compared to *hlyA* from other clade C strains, most likely due to recombination. Analysis of the *hlyCBD* genes revealed a similar phylogenetic relationship between the clade C and clade A/B strains (see Fig. S4A in the supplemental material). To examine amino acid variation in HlyA further, we mapped the location of the changes and showed the majority lie outside known HlyA functional domains (see Fig. S5 in the supplemental material). The impact of these sequence changes on hemolysin activity was also examined by cloning the *hlyCABD* locus from strains representative of this clustering (S65EC, HVM277, and S115EC) into the expression vector pSU2718 (∼15 copies per cell [63]) to generate plasmids pHly^S65EC^, pHly^HVM277^, and pHly^S115EC^. Transformation of these plasmids into the K-12 strain MG1655 revealed that the recombinant strains possessed a similar hemolytic profile to their respective parent strain; i.e., MG1655 harboring pHlyA^S65EC^ or pHlyA^S115EC^ was significantly more hemolytic than MG1655 harboring pHlyA^HVM277^ (Fig. 4B). Together, these data suggest that the polymorphisms in the *hlyCABD* coding sequences, together with sequence variation in the leader transcript region, account for the differential hemolytic activity of clade C versus clade A/B strains.

10.1128/mBio.02248-19.5 FIG S4 Variation in *hlyCBD* genes among ST131 isolates. (A) Phylogenetic trees of *hlyCBD* genes. Phylogenetic analysis of the *hlyCBD* genes with sequences retrieved from assemblies of hemolysin-positive ST131 strains and aligned with ClustalO. Maximum likelihood trees were generated from these alignments using RaxML, with 1,000 bootstraps. Strain names are color coded according to their clade designation: red, clade A; orange, clade B; green, clade C. The scale indicates the number of substitution SNPs, and asterisks denote branches with supported bootstrap values of >90%. (B) Phenotypic analysis of red blood cell (RBC) hemolysis conferred by (i) wild-type HVM277 and HVM2044 strains and (ii) MG1655 harboring pHly^HVM277^, pHly^HVM2044^, or the vector control plasmid pSU2718. The data support the interpretation that the nonsynonymous SNP difference in *hlyB* (P538L) is associated with reduced hemolytic activity. Download FIG S4, TIF file, 2.5 MB. Copyright © 2019 Nhu et al. 2019 Nhu et al. This content is distributed under the terms of the Creative Commons Attribution 4.0 International license.

10.1128/mBio.02248-19.6 FIG S5 Overview of amino acid sequence variation in the HlyA variants from S65EC, HVM277, and S115EC. (A) Schematic view of HlyA protein sequence with specific domains indicated (black, transmembrane and pore forming domain; green, glycine-rich repeat units; orange, acylated lysine; opal, secretion signal [sec]). The positions of amino acid variations relative to the sequence of HlyA from S65EC are shown as the dark blue vertical line underneath. (B) Documentation of amino acid variations and their corresponding positions in S65EC, HVM277, and S115EC compared to HlyA from prototype UPEC strains UTI89 and CFT073. Dots represent the same amino acid shown in S65EC. Download FIG S5, TIF file, 1.8 MB. Copyright © 2019 Nhu et al. 2019 Nhu et al. This content is distributed under the terms of the Creative Commons Attribution 4.0 International license.

We previously characterized the clade B strain HVM277 as a low-level hemolysin producer (32). Intriguingly, while this strain possessed an identical 1.616-kb leader sequence and similar *hlyC/hlyA* transcript levels to the other clade B strains HVM2044 and HVM52, their hemolytic activities differed significantly (Fig. 2A). Closer analysis revealed only 1 nonsynonymous SNP difference in *hlyB* (P538L) between HVM277 (∼29% hemolysis) and HVM2044 and HVM52 (∼4% hemolysis) (Fig. S4A). In addition, Western blot analysis employing a HlyA-specific antibody revealed that although HlyA could be found in the cell pellets of HVM2044, no HlyA could be detected in the supernatant (Fig. 2D). Furthermore, MG1655 harboring pHlyA^HVM2044^ was less hemolytic than MG1655 harboring pHlyA^HVM277^, indicated by the smaller zone of hemolysis on blood agar (Fig. S4B). Taken together, the data suggest this nonsynonymous mutation in the *hlyB* gene in HVM2044 and HVM52 may impair the HlyA export machinery, and thus contribute to the weak hemolytic activity observed in these strains.

### Acquisition of the hemolysin locus in ST131 is linked to two independent insertion events.

The concordance between the sequence of the *hlyCABD* locus, hemolytic activity, and strain phylogeny prompted us to examine the genetic location of the hemolysin genes in our strain set. Analysis of the draft assembled Illumina sequence data from the clade B strains HVM52 and HVM277 revealed the *hlyCABD* genes are located on a single contig that spans a GI integrated at *leuX*-tRNA (GI-HVM52-*leuX* and GI-HVM277-*leuX*, respectively) (see Fig. S6 in the supplemental material). We were unable to assemble a single contig that could define the genomic location of the *hlyCABD* locus in any of the clade C2 strains, and thus we employed PacBio SMRT sequencing and used this together with our Illumina data to generate a hybrid assembly and complete genome sequence of the hemolysin-positive clade C2 strain S65EC. Overall, the S65EC genome comprises a chromosome containing 5,187,769 nucleotides and a large IncF plasmid (pS65EC, 146,792 bp, F1:A-:B23) (see Fig. S7 in the supplemental material). Analysis of the *hlyCABD* locus in S65EC revealed it is located within a GI integrated at *pheU*-tRNA (GI-S65EC-*pheU* [Fig. S6]). Although GI-S65EC-*pheU* shares many common features with GI-HVM52-*leuX* and GI-HVM277-*leuX*, including genes encoding P and F17 fimbriae and the Cnf1 toxin (Fig. S6), the sequence variation and different genomic location of the *hlyCABD* genes suggest they were acquired independently by clade A/B and clade C ST131 strains.

10.1128/mBio.02248-19.7 FIG S6 Genomic island locations of *hlyCABD* in clade B strains (HVM277 and HVM52) and clade C strain (S65EC). The hemolysin operon is located in GI-*leuX* and GI-*pheU* in clade B and clade C strains, respectively. In these three strains, *hly* operons are found together with cytotoxic necrotizing factor 1 gene (*cnf-1*), intact *pap* operon, and F17 fimbrial gene cluster (indicated as red), flanked by mobile elements (including insertion sequences and transposons) indicated by blue. Hypothetical proteins are shown in black. The figure was drawn to scale. Download FIG S6, TIF file, 1.5 MB. Copyright © 2019 Nhu et al. 2019 Nhu et al. This content is distributed under the terms of the Creative Commons Attribution 4.0 International license.

10.1128/mBio.02248-19.8 FIG S7 Comparative genomics of S65EC. (A) Comparative genomics between S65EC (middle) and two prototypic ST131 clade C strains EC958 (top) and JJ1886 (bottom) showed the flexibility of ST131 genomes. Mobile elements (prophages [Phi] and genomic islands [GI]) and regions of interest (ROI, ST131 region of different) are indicated by blue boxes. S65EC was isolated from the urine of an elderly patient in a nursing home in Australia in 2009. Previous studies showed that S65EC belongs to ST131 clade C2 and is resistant to fluoroquinolones due to mutations in *gyrA* and *parC* genes, and harbors *bla*_CTX-M-15_ (9, 10). (B) Location of *bla*_CTX-M-15_ in S65EC and pEC958. Antibiotic-resistant genes are indicated as red and mobile elements (including insertion sequences and transposons) as blue. Whole-genome sequencing of S65EC revealed that unlike in the prototypic ST131 strain EC958, *bla*_CTX-M-15_ has been integrated into the S65EC chromosome via a Tn*3* transposable element. Upstream of *bla*_CTX-M-15_ is the IS*Ecp1* insertion element, which acts as its promoter and drives the transcription of *bla*_CTX-M-15_ as reported previously (105). In addition, a macrolide-resistant gene cluster (*mphA*, *mrx*, and *mphR*) is inserted downstream of the *bla*_CTX-M-25_ by IS*26*. Download FIG S7, TIF file, 1.0 MB. Copyright © 2019 Nhu et al. 2019 Nhu et al. This content is distributed under the terms of the Creative Commons Attribution 4.0 International license.

### Development of a genome-wide screen for UPEC mutants with altered hemolysin activity.

To expand our analyses and to identify uncharacterized mechanisms by which hemolysin expression is regulated, we devised a forward genetic screen to define the set of genes involved in hemolysin production. We generated a saturated transposon mutant library in S65EC using a mini-Tn*5* transposon and screened the library on sheep blood agar to identify mutants significantly altered in their hemolytic phenotype (i.e., a decrease or increase in the zone of hemolysis compared to the parent strain). In total, ∼177,000 mini-Tn*5* mutants were screened, from which there were 77 nonhemolytic mutants, 34 mutants with reduced hemolytic activity, and 22 mutants with increased hemolytic activity. These mutants were pooled according to their hemolysis phenotype and examined by TraDIS to enable *en masse* identification of the insertion sites that led to altered hemolysin activity. In addition, colonies from the library of 177,000 transposon mutants were also pooled and analyzed by TraDIS as the input pool, thus enabling us to accurately determine the overall insertion frequency and coverage of our miniTn*5* mutant library.

### Identification of genes associated with hemolysin production.

**FIG 5:**
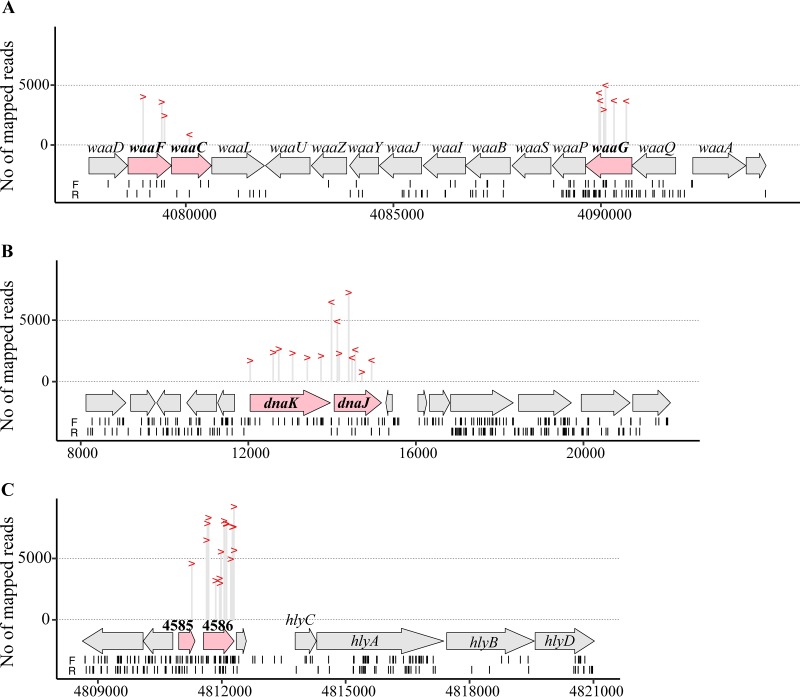
TraDIS identified novel genes involved in hemolysin production. Location and number of reads mapped to each insertion site within the (A) *waa* locus, (B) *dnaKJ*, and (C) the upstream region of *hlyC*. Each insertion site is represented by “>” (mini-Tn*5* insert with the promoter of the chloramphenicol resistance gene orientated in the forward direction) or “<” (mini-Tn*5* insert with the promoter of the chloramphenicol resistance gene orientated in the reverse direction). Arrows represent the coding sequences. Locations of insertion sites in the input pool are shown by short vertical lines underneath, with “F” and “R” indicating mini-Tn*5* inserts with the promoter of the chloramphenicol resistance gene orientated in the forward or reverse direction, respectively.

TraDIS analysis of the input pool from 1,307,913 sequence reads showed that these reads mapped to 75,330 unique insertion sites in the S65EC genome (see Fig. S8 in the supplemental material). This equated to approximately one mini-Tn*5* insertion every 70 bp of the genome, demonstrating broad coverage of our screen. Analysis of the three output pools from 444,245 sequence reads identified 122 insertion sites, broken down into 67 insertion sites from the nonhemolytic pool, 33 insertion sites from the reduced-hemolytic pool, and 22 insertion sites from the increased-hemolytic pool, respectively (Fig. S8). These insertion sites were further localized to 17 genes (Table 1; Fig. 5), of which seven had a known role in hemolysin production (*hlyCABD*, *tolC*, *rfaH*, and *hns*). A role for two of the genes (*dnaK* and *rne*) could not be verified due to inability to generate defined mutants, while the other genes were novel or have not been well studied with respect to their role in hemolysin production, and thus we focused the remainder of our study on their characterization.

**TABLE 1:** Genes impacting hemolysin activity identified by TraDIS

Gene name	No. of inserts	No. of reads	Hemolysin activity in^*a*^:		HlyA expression by defined mutants in^*b*^:		Product(s)
			Tn*5* mutants	Defined mutants	Supernatant	Cell lysate	
*hlyCABD* operon							
*hlyC*	3	2,878	None	ND	ND	ND	Acyltransferase HlyC
*hlyA*	30	38,070	None	None	None	None	Hemolysin A
*hlyB*	2	1,951	None	ND	ND	ND	ATP-binding protein HlyB
*hlyD*	10	14,745	None	ND	ND	ND	Membrane fusion protein HlyD
Transporter							
*tolC*	1	9,586	None	None	None	Yes	Outer membrane protein TolC
Regulators							
*hns*	3	6,915	Increased	ND	ND	ND	Global regulator H-NS
*rfaH*	2	8,395	Reduced	ND	ND	ND	Transcriptional antiterminator RfaH
LPS inner core biosynthesis							
*rfaE*	2	9,289	None	None	None	Yes	Fused heptose 7-phosphate kinase/ heptose 1-phosphate adenyltransferase
*waaC*	1	849	None	None	None	Yes	ADP-heptose:LPS heptosyltransferase I
*waaF*	3	10,017	None	None	None	Yes	ADP-heptose:LPS heptosyltransferase II
*wag*	6	23,276	None	Reduced	None	Yes	Glucosyltransferase I
DnaKJ chaperones							
*dnaK*	5	11,329	Reduced	ND	ND	ND	Chaperone
*dnaJ*	4	16,622	No/reduced	None	None	Yes	Chaperone
Other genes							
*acrR*	2	7,709	Reduced	As wt	ND	ND	Repressor AcrR for AcrAB in AcrAB-TolC multidrug efflux pump
*rne*	1	3,757	Reduced	ND^*c*^	ND	ND	Ribonuclease E
S65EC_04585	1	4,576	Increased	As wt^*d*^	ND	ND	Putative thiosulfate reductase cytochrome *b* subunit YdhU
S65EC_04586	13	81,439	Increased	As wt^*d*^	ND	ND	Putative sulfite oxidase subunit YedY

a Hemolytic activity compared to the wild type. None, not detected; ND, not done; wt, wild type.

b Hemolysin expression was detected by Western blotting using HlyA-specific antibody.

c Unable to generate defined mutant due to the essentiality of the gene.

d Refers to hemolytic activity of the mutant with the chloramphenicol resistance gene cassette removed. The mutant with the *cat* gene cassette present showed increased hemolysin activity due to the read-through from the *cat* promoter.

10.1128/mBio.02248-19.9 FIG S8 Transposon insertion sites in the input and output pools. Shown are the numbers of sequencing reads per insertion site (represented by a dot) in the input, no-hemolytic, reduced-hemolytic, and increased-hemolytic pools along the S65EC genome. Genes with transposon insertions are annotated. Dots without annotation represent insertions within intergenic regions. Download FIG S8, TIF file, 2.7 MB. Copyright © 2019 Nhu et al. 2019 Nhu et al. This content is distributed under the terms of the Creative Commons Attribution 4.0 International license.

### Disruption of LPS core biosynthesis prevents hemolysin secretion.

**FIG 6:**
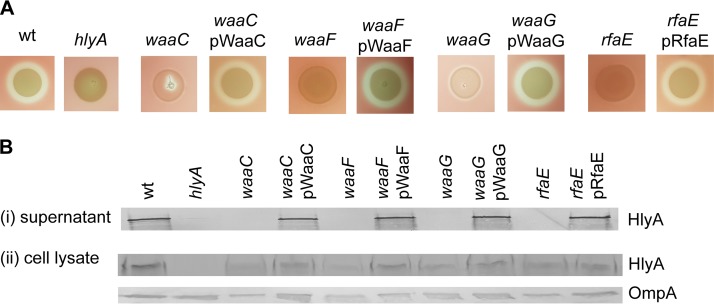
Genes involved in LPS inner core biosynthesis contribute to hemolytic activity. (A) Hemolysin activity of S65EC, S65*hlyA*, and defined LPS inner core mutants and their complemented strains on sheep blood agar. Mutation of genes in LPS inner core biosynthesis abolished hemolysin activity, while complementation restored this phenotype. (B) Western blot analysis of HlyA performed using concentrated supernatant (i) or whole-cell lysates (ii) prepared from S65EC, S65*hlyA*, and defined LPS inner core mutants and their complemented strains. Bacterial cell lysates were analyzed for OmpA expression as a loading control. Hemolytic assays and immunoblots in panels A and B, respectively, are representative of three independent experiments.

Our TraDIS analysis identified 12 unique insertion sites in four genes involved in LPS inner core biosynthesis; *waaC* (from the nonhemolytic pool), and *rfaE*, *waaF* and *waaG* (from the reduced-hemolytic pool) (Fig. 5A; Table 1). To validate the TraDIS data, we generated defined mutants for each gene via λ-Red mediated homologous recombination. Compared to the parent S65EC strain, all four mutants possessed an abolished/reduced hemolytic activity profile that was restored to wild-type level by in *trans* complementation with the corresponding gene (Fig. 6A). Next, we tested if the mutation of these core LPS biosynthesis genes affected hemolysin secretion by examining the level of HlyA in whole-cell lysates and the culture supernatant of each mutant by Western blotting. We showed that mutation of each of these genes abolished hemolysin secretion, and this could be restored by complementation (Fig. 6B). In contrast, HlyA was detected in total cell lysates prepared from each mutant (Fig. 6B), demonstrating that disruption of LPS inner core biosynthesis did not affect production of HlyA, but impaired its secretion.

### The DnaK and DnaJ chaperones are required for hemolysin secretion.

**FIG 7:**
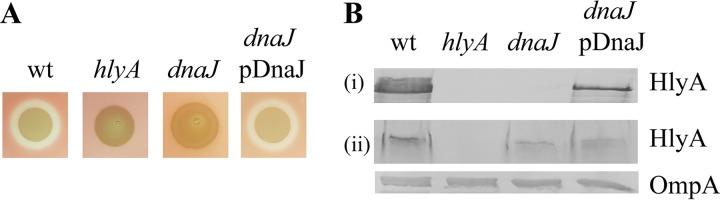
DnaJ required for hemolysin secretion. (A) Hemolysin activity of S65EC, S65*hlyA*, and the S65*dnaJ* defined mutant and its complementation on sheep blood agar. Disruption of *dnaJ* abolished hemolysin activity, while complementation restored this phenotype. (B) Western blot analysis of HlyA performed using concentrated supernatant (i) or whole-cell lysates (ii) prepared from S65EC, S65*hlyA*, S65*dnaJ* and its complementation grown to late log phase. Bacterial cell lysates were analyzed for OmpA expression as a loading control. Hemolytic assays and immunoblots in A and B, respectively, are representative of three independent experiments.

The *dnaK* (five unique insertion sites) and *dnaJ* (four unique insertion sites) genes were identified in the pool of mutants with reduced hemolytic activity (Fig. 5B; Table 1). DnaK is the major Hsp70 class chaperone in the E. coli cytosol, and together with its cochaperone DnaJ and regulator GrpE it plays a key role in the folding of nascent polypeptides (64–66). Given that a *dnaK* null mutant displays growth defects (67, 68) and the complementation of *dnaK* on a multiple-copy plasmid has been shown to be unstable (69), we confirmed our TraDIS data by mutating *dnaJ*, the second gene in the *dnaKJ* operon. This strain, designated S65EC*dnaJ*, was nonhemolytic, and hemolysis was restored by complementation with a plasmid containing the *dnaJ* gene (pDnaJ [Fig. 7A]). Western blot analyses of supernatant and whole-cell lysate fractions revealed that hemolysin was produced by S65EC*dnaJ*, but not secreted (Fig. 7B).

### Hemolysin production increases when a strong promoter is inserted upstream of *hlyCABD*.

**FIG 8:**
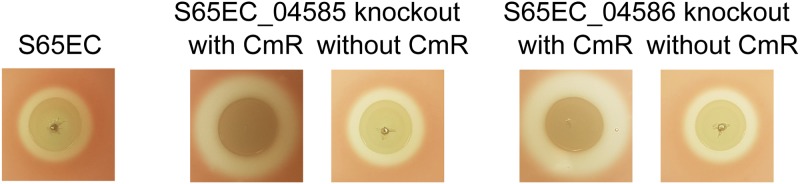
Overexpression of hemolysin by a strong promoter inserted upstream of the 1.616-kb leader sequence. Shown are phenotypes of defined S65EC mutants following insertional inactivation of S65EC_04585 and S65EC_04586 and growth on sheep blood agar. Compared to the wild type, disruption of S65EC_04585 and S65EC_04586 led to increased hemolysin activity due to read-through from the chloramphenicol resistance gene cassette (CmR) promoter. When this CmR cassette was removed, the defined mutants expressed hemolysin as the same level as the wild type. Hemolytic assays are representative of three independent experiments.

Previous studies have shown that the promoter of the chloramphenicol resistance gene in our mini-Tn*5* transposon can drive the transcription of a downstream gene if the insertion position is favorable (70, 71). We therefore predicted that mini-Tn*5* insertions upstream of *hlyC* would be associated with increased hemolysin activity. TraDIS analysis of our input pool revealed five mini-Tn*5* insertions within the long *hlyCABD* leader transcript (Fig. 5C). Although all of these insertions introduced a promoter orientated in the same direction as the *hlyCABD* genes, none of the mutants were identified in the increased-hemolytic pool. In contrast, we identified 14 unique mini-Tn*5* insertions in the coding sequences upstream of this region in the increased-hemolytic pool: one insertion within *ydhU* (S65EC_04585, encodes a putative thiosulfate reductase cytochrome *b* subunit) and 13 insertions within *yedY* (S65EC_04586, encodes a putative sulfite oxidase subunit) (Fig. 5C; Table 1). These mini-Tn5 insertions were all located upstream of the JUMPStart sequence, with the chloramphenicol resistance gene promoter pointing in the direction of the downstream *hlyCABD* genes (Fig. 5C). To show that this increase in hemolytic activity was not due to specific disruption of the *ydhU* and *yedY* genes, we mutated these genes in S65EC using λ-Red recombination (with the chloramphenicol resistance gene cassette in the same direction of the *hlyCABD* genes and subsequent removal of the cassette using an FLP recombinase). Both mutants possessed increased hemolytic activity when the chloramphenicol resistance gene was present, but this returned to the wild-type level upon removal of the cassette (Fig. 8). Thus, we conclude that insertion of a strong promoter upstream of the *hlyCABD* genes can enhance transcription of the *hlyCABD* genes, but this occurs most favorably when the JUMPStart site and long 1.616-kb leader sequence remain intact.

## DISCUSSION

Epidemiological studies show that *hlyA* prevalence is associated with UPEC strains that cause severe UTI (16, 17). However, the level of hemolysin expression and its impact on virulence are variable and often strain specific (33, 34). Here, we investigated the prevalence of the *hlyA* gene in 83 of the most common E. coli STs and then performed a detailed analysis of sequence variation focusing on the globally dominant multidrug-resistant ST131 clone. Using a combination of bioinformatics and functional analyses, we examined the relationship between hemolytic activity and genomic variation in the ST131 lineage. Finally, we also applied a large-scale forward genetic screen to identify new genes involved in hemolysin production.

Within the ST131 clone, HlyA-positive clade C2 strains were more hemolytic than clade A and B strains, and this corresponded with increased transcription of the *hlyCABD* genes. Several studies have demonstrated that the production of hemolysin leads to enhanced virulence (31, 32, 34, 40, 72). In the rat peritonitis model, hemolysin production correlates with increased invasiveness and lethality (33, 34, 59). In the mouse UTI model, hemolysin production leads to shedding of uroepithelial cells, increased inflammation, and enhanced hemorrhaging during the early phase of infection (47). In addition, fine-tuning of hemolysin expression can alter the outcome of UTI, ranging from persistence to acute infection (31). We also recently showed that high levels of hemolysin production contribute to enhanced bladder colonization during experimental UTI, with this linked to rapid macrophage cell death that limits host-protective cytokine production (32). Our findings in this study demonstrate the most multidrug-resistant clade C2 ST131 strains also possess the strongest hemolytic activity, revealing a new link between enhanced virulence and multidrug resistance.

The region upstream of the *hlyCABD* coding sequence plays a role in the regulation of hemolysin expression (34, 39, 41, 45). We mapped the promoter of the *hlyCABD* genes in S65EC and identified a long 1.616-kb leader transcript that is conserved in all HlyA-positive ST131 strains. This 1.616-kb long 5′ leader sequence contains a high AT content (64.4%), which could increase stability of the *hlyCABD* mRNA and therefore enhance translation as reported previously for other AU-rich 5′ leader mRNA sequences (73, 74). Our results are in line with a study from Cross et al., who also showed that the 2-kb upstream region of *hlyC* is involved in the regulation of hemolysin expression in UPEC strain LE2001 (39). In the reference UPEC strain J96, the leader transcript is shorter and lies 462 to 464 bp upstream of the *hlyC* start codon (75). This transcription start site in J96 was mapped from a plasmid containing the cloned *hlyCABD* genes (76), so we cannot exclude the possibility that the differences are due to the plasmid versus chromosomal location of the *hlyCABD* genes. Sequence analysis of this long leader sequence and the *hlyCABD* genes, together with their chromosomal location, suggests their acquisition in ST131 has occurred independently in clade A/B versus clade C strains.

The combination of high-throughput genome-wide random transposon mutagenesis and TraDIS represents a powerful tool for understanding complex phenotypes (70, 77–80). By screening large numbers of transposon mutants under stringent selective conditions, it is possible to simultaneously identify all of the genes involved in a given pathway. In this study, we screened ∼177,000 transposon mutants for altered hemolytic activity. Notably, by performing TraDIS analysis on the input pool, we were also able to verify high coverage of our mutant library and thus demonstrate the comprehensiveness of our screen. In total, we confirmed a role for 13 genes in hemolysin production. This included the previously characterized *hlyCABD* genes, the outer membrane transporter *tolC* and the transcriptional antiterminator *rfaH*, where in all cases the mini-Tn*5* insertion led to the abolition or severe reduction of hemolysin activity. The role of these genes in hemolysin production and secretion is well established (20, 23, 43, 45, 76, 81); hence their detection validated our screen. In line with previous reports (37, 38, 82), we also confirmed the role of H-NS as a repressor of hemolysin.

The identification of four core LPS biosynthesis genes in our screen provides very strong evidence that the secretion of hemolysin is intrinsically tied to LPS biosynthesis. Although early studies also demonstrated this connection, they were performed in E. coli K-12 mutants with the *hlyCABD* genes introduced in *trans* on a plasmid (83–85), thus possibly masking subtle phenotypic changes due to high levels of hemolysin expression. Our TraDIS screen, performed in the completely sequenced S65EC clade C2 ST131 strain, showed that mutants containing deletions in *rfaE*, *waaC*, and *waaF* were unable to lyse red blood cells, while a *waaG* mutant caused reduced hemolytic activity. With respect to function, the *rfaE* gene encodes an enzyme required for heptose synthesis (86), while the *waaF* and *waaC* genes encode enzymes involved in synthesis of the inner LPS core oligosaccharide, where they transfer the first and second heptoses onto the Kdo_2_-lipid A (87, 88). The *waaG* gene encodes an enzyme involved in synthesis of the outer LPS core and functions by adding the first glucose to the second heptose residue (89). We hypothesize that interaction between TolC and the LPS core is critical for hemolysin secretion, as has been suggested previously (85), thus explaining the subtle difference in the phenotype of our *waaG* versus *rfaE*, *waaC*, and *waaF* mutants. We note that hemolysin has also been shown to form a complex with LPS (90–92), and the binding of LPS enhances the stability of the toxin and reduces HlyA self-aggregation. In addition, due to its negative charge, it has been suggested that LPS may provide a reservoir of calcium, an important cofactor required for HlyA activity (93). Thus, we cannot rule out other mechanisms by which disruption of the LPS core might affect hemolysin secretion and activity.

Our study also revealed the involvement of the DnaK-DnaJ chaperones in controlling hemolysin activity. DnaK (and DnaJ) function as ATP-dependent Hsp70 chaperones that play a critical role in the folding of nascent polypeptides and the refolding of damaged proteins in the cytoplasm (64). The activity of DnaKJ involves the regulator GrpE (64). DnaJ binds to nonnative substrate proteins, and transfers them to ATP-bound DnaK. ATP hydrolysis, elevated by DnaJ, enhances interaction of the DnaK-substrate complex. After ATP hydrolysis, DnaJ is released, and GrpE binds to the ATPase domain of DnaK to catalyze the formation of ADP, resulting in release of the substrate for folding or transfer to other chaperones (64–66). Previous studies have shown that DnaK interacts with ∼700 proteins, the majority of which are cytosolic and prone to aggregation during and after initial folding (64). Although the precise molecular mechanism by which DnaK-DnaJ chaperones interact with HlyA remains unclear, it has been demonstrated that folded substrates are not effectively secreted through the type 1 secretion system (94, 95). Thus, we suggest that DnaK/DnaJ contribute to efficient secretion of HlyA by maintaining its unfolded state or slowing down its folding rate in the cytoplasm.

Several genes were identified in our screen but could not be verified based on the phenotypic characterization of a defined mutant, including *acrR*, *ydhU*, *yedY*, and *rne* (Table 1). While mini-Tn*5* insertions in *ydhU* and *yedY* led to enhanced hemolytic activity, we showed this was not due to mutation of the respective genes, but rather due to favorable insertion of a strong promoter upstream of the *hlyCABD* genes. In the case of the *rne* gene, which encodes RNase E, we were unable to generate a defined mutant to confirm our TraDIS data despite multiple attempts. The *rne* gene has been described as essential in another study (96), offering some explanation for the difficulty in generating this mutant. Finally, we previously identified the *cof* gene as a regulator of secreted HlyA in CFT073 (which belongs to ST73), where mutation of the *cof* gene led to reduced hemolysin production (30). In the current screen performed on ST131 strain S65EC, we did not identify insertions in *cof* that resulted in reduced hemolysin activity (despite nine insertions in this gene in the input pool), suggesting that the role of *cof* in hemolysin regulation may be strain-specific.

Factors that negatively regulate hemolysin expression have been reported, including H-NS and the stress response regulator CpxR. Disruption of *hns* increases the expression of several virulence factors in E. coli, including hemolysin (37, 38, 82, 97). CpxR has been shown to bind to the *hlyCABD* promoter and repress *hlyA* transcription (31). In this study, we also screened for mutants that possessed enhanced hemolysin activity and confirmed the role of *hns* as a repressor of hemolysin. However, we did not identify insertions in *cpxR* that resulted in enhanced hemolysin activity, even though there were 16 unique insertion sites in this gene in the input pool. This could be due to the difference in strains used in the two studies (UTI89, ST95, versus S65EC, ST131). We also identified 14 independent insertion sites immediately upstream of the 1.616-kb *hlyCABD* leader sequence that caused enhanced hemolytic activity. Precise mapping of these insertions by TraDIS revealed they all contained the *cat* promoter pointing toward the *hlyCABD* operon, and we demonstrated that these insertions lead to an increase in hemolysin expression caused by read-through from the *cat* promoter, as reported in other studies (70, 71). Intriguingly, we did not identify mini-Tn*5* insertions within the 1.616-kb *hlyCABD* leader sequence that caused enhanced hemolytic activity, even though such insertions were present in the input pool, suggesting there are multiple features within this untranslated mRNA leader sequence (including the *ops* element and JUMPStart sequence) that are critical for transcription of the *hlyCABD* genes.

In summary, this work has discovered important new features of hemolysin regulation and variation by studying its biology in the context of the well-defined genealogy of the globally disseminated multidrug-resistant ST131 clone. Our study revealed that nucleotide sequence variation in the hemolysin locus (including its long 5′ leader sequence) accounts for differential gene transcription, as well as altered hemolysin secretion and activity, and these differences are underpinned by the location of this locus within diverse horizontally acquired genomic islands. Furthermore, our application of a large-scale forward genetic screen has defined new chaperone and core LPS components that are required for secretion of this important UPEC toxin.

## MATERIALS AND METHODS

### Ethics approval.

All experiments using primary human cells were approved by the University of Queensland Medical Research Ethics Committee (2013001519).

Key experimental procedures used in the study are listed below. Extended experimental methods, including (i) generation of human monocyte-derived macrophages, (ii) *in vitro* infection assays, (iii) whole-genome sequencing and analysis, (iv) transposon mutagenesis and transposon-directed insertion site sequencing, (v) targeted gene mutation and complementation, (vi) generation of plasmids containing variant *hlyCABD* alleles, and (vii) sample preparation for Western blotting, are provided in Text S1 in the supplemental material.

10.1128/mBio.02248-19.1 TEXT S1 Supplemental materials and methods. Download Text S1, DOCX file, 0.03 MB. Copyright © 2019 Nhu et al. 2019 Nhu et al. This content is distributed under the terms of the Creative Commons Attribution 4.0 International license.

### Strains and bacterial growth conditions.

The E. coli ST131 strains used in this study have been described previously (9). Bacterial strains were grown at 37°C on solid or in liquid lysogeny broth (LB) medium unless otherwise indicated. Chloramphenicol (30 μg/ml) or kanamycin (50 μg/ml) was added as required.

### Hemolysis assays.

Hemolysis assays were performed on blood agar or in liquid culture, essentially as described previously (98) but with minor modifications. Briefly, the zone of hemolysis was measured after spotting 5 μl of filtered supernatant from a bacterial overnight culture onto blood agar (LB agar containing 5% fresh sheep red blood cells and 10 mM CaCl_2_) and incubating at 37°C for 16 to 24 h. In addition, the level of hemolysis was quantitated by incubating approximately 10^7^ CFU/ml of bacteria for 3 h in LB broth containing 5% sheep blood and 10 mM CaCl_2_ and measuring the released hemoglobin at a wavelength of 540 nm compared to the released hemoglobin of blood in water alone.

### Sequencing data, sequence alignment, and phylogenetic analyses.

Assemblies of E. coli strains belonging to ST69, ST73, ST95, and ST131 were downloaded from EnteroBase in July 2018 (https://enterobase.warwick.ac.uk). In addition, approximately 100 sequence assemblies were randomly chosen from each of the top 83 E. coli sequence types in the E. coli collection on EnteroBase, resulting in a collection of 8,247 assemblies downloaded in January 2019. The prevalence of the *hlyA* gene encoding hemolysin or *hlyCABD* was determined in these strains from EnteroBase and 95 in-house ST131 strains (9) using BLASTn (99) against the *hlyA* gene or *hlyCABD* from the CFT073 genome (AE014075.1), with the cutoff at 90% nucleotide sequence conservation and 80% length coverage.

To compare sequence variation, the *hlyCABD* operon, as well as individual genes, was extracted from the 14 hemolysin-positive ST131 strains from previous studies (9, 10). Alignment was performed with ClustalO (100), from which maximum likelihood trees were generated using RaxML v.7.2.8, with the general time-reversible (GTR) GAMMA model of among-site rate variation (ASRV) (101). The robustness of the trees was tested with 1,000 bootstraps. Trees were visualized and edited using FigTree v1.3.1.

### RNA extraction, qRT-PCR, and 5′ RACE.

Total bacterial RNA was extracted from late-log-phase bacterial cultures (optical density at 600 nm [OD_600_] = 0.9 to 1) in LB broth using the RNeasy minikit (Qiagen) as per the manufacturer’s instructions. Total mRNA was converted into cDNA using random hexamer primers and SuperScript III reverse transcriptase (Invitrogen, Life Technologies). Quantitative reverse transcription-PCR (qRT-PCR) was performed for the *hlyC* and *hlyA* genes using the ABI SYBR green PCR master mix on the ViiA 7 real-time PCR system (Life Technologies) with primers listed in Table S1 in the supplemental material. The relative transcript level of each gene was compared to the corresponding gene in HVM2044; fold change was calculated by the threshold cycle (2^−ΔΔ^*^CT^*) method (102) using *gapA* as an endogenous control (103).

10.1128/mBio.02248-19.10 TABLE S1 Primers used in this study. Download Table S1, DOCX file, 0.02 MB. Copyright © 2019 Nhu et al. 2019 Nhu et al. This content is distributed under the terms of the Creative Commons Attribution 4.0 International license.

The transcriptional start site of the *hlyCABD* genes in S65EC was identified using the 5′ RACE system (Qiagen) according to the manufacturer’s instructions. cDNA specific for *hlyC* was synthesized from total RNA using SuperScript III reverse transcriptase (Invitrogen, Life Technologies) with specific primers *hlyC_GSP1* and *hlyC_GSP12* (Table S1). These PCR amplicons were sequenced using the BigDye Terminator v3.1 Cycle Sequencing kit (Life Technology) with the primer *hlyC_GSP14* (Table S1).

### Western blotting.

Bacterial cell pellets were harvested from the late-log-phase cultures and resuspended in TCU buffer (1:100 [vol/vol]) (104). The supernatants were sterilized by filtering through a 0.22-μm-pore membrane, and secreted proteins were concentrated 100 times using ammonium sulfate 60% (wt/vol) overnight at 4°C. Detection of HlyA in secreted proteins and the cell lysates was performed with specific monoclonal antibody H10 against HlyA as described previously (30).

### Accession number(s).

All sequence data for this study have been deposited under BioProject no. PRJNA517996. The sequences for the S65EC chromosome and plasmid pS65EC are available in the NCBI GenBank database under accession no. CP036245 and CP036244, respectively. The raw PacBio sequence reads have been deposited in the Sequence Read Archive (SRA) under accession no. SRR8535518. The TraDIS reads have been deposited in the SRA under accession no. SRR8535515 to SRR8535517.
