# Supplementary material for: Complex Multilevel Control of Hemolysin Production by Uropathogenic Escherichia coli
Source: mBio. 2019 Oct 1;10(5):e02248-19. doi: 10.1128/mBio.02248-19 (PMC6775461; doi:10.1128/mBio.02248-19)
Supplement: TEXT S1 [file mBio.02248-19-s0001.docx]

**Supplementary materials and methods**

**Generation of human monocyte-derived macrophages (HMDM)**

All experiments using primary human cells were approved by The University of Queensland Human Research Ethics Committee. Monocytes were isolated from buffy coats of healthy donors (kindly provided by the Australian Red Cross) by density gradient centrifugation, followed by positive selection for CD14^+^ using magnetic-activated cell sorting (Miltenyi Biotech). Human monocytes were differentiated into macrophages as previously described (1), and each experiment used cells from a different donor.

***In vitro* infection assays**

HMDM were seeded overnight at a density of 8 x 10^4^ cells/well in a 96 well plate in RPMI media containing 10% fetal bovine serum (Gibco, Life Technologies), 2 mM L-glutamine (Gibco, Life Technologies) and 1 x 10^4^ U/mL recombinant human CSF-1 (Chiron). Overnight cultures of UPEC strains were pelleted, washed twice with Dulbecco’s phosphate buffered saline (Gibco, Life Technologies), resuspended in RPMI and diluted to the same optical density, after which HMDM were infected with the indicated multiplicity of infection (MOI). MOIs were confirmed by counting colony-forming units after serial dilution. At 1 h post-infection, 200 μg/mL gentamicin (Life Technologies) was added to prevent extracellular bacterial growth. At 2 h post-infection, cell culture supernatants were replaced with fresh RPMI medium containing 20 μg/mL gentamicin. Cell death was recorded using LDH release assays, as previously described (2). In brief, cell culture supernatants were collected at 8 h post-infection, centrifuged at 500 g for 5 min, and then analyzed for LDH release using CytoTox96 non-radioactive Cytotoxicity Assay kits (Promega).

**Whole genome sequencing and analysis**

Genomic DNA from S65EC was extracted using Ultraclean**^®^** Microbial DNA Isolation Kit (MO BIO), and subjected to sequence on a PacBio RS II sequencing instrument using 3 SMRT cells, a 10 kb insert library and the P6-C4 sequencing chemistry. *De novo* genome assembly was performed using PacBio's SMRT Portal (v2) and the hierarchical genome assembly process (HGAP v2.0) with default settings and a seed read cut-off length of 5 kb. Assembly resulted in 2 contigs, a chromosome and one plasmid. Additional genome sequence polishing was required to resolve single nucleotide insertion and deletion errors associated with homopolymer tracts. Illumina sequence data for S65EC was aligned to the draft genome using bwa version: 0.7.12-r1039 (3) and a corrected consensus was called using Pilon version 1.21 with default parameters and the ‘--fix indels’ flag (4).

The complete genome sequence of S65EC was annotated using Prokka (5) and insertion sequence (IS) annotation was done with ISFinder (<https://www-is.biotoul.fr/>). Annotation of CDS and IS was then curated manually. Phage and genomic islands were identified with PHAST (6) corrected manually. Comparative genomics of were performed using BLAST (7), BRIG (8), ACT (9) and Easyfig (10).

**Transposon mutagenesis and transposon directed insertion site sequencing (TraDIS)**

A transposon mutant library was generated for *hlyCABD*-positive strain S65EC as previously described (11). Briefly**,** transposomes generated with EZ-Tn*5* transposase (Epicenter Biotechnologies) and a miniTn*5* transposon containing chloramphenicol resistance gene (*cat*) were electroporated into electro-competent S65EC cells. Transformed cells were recovered in SOC media for 2 hours before being plated onto LB-chloramphenicol agar containing 5% sheep blood and 10 mM CaCl_2_, and incubated at 37°C for 18 hours. All recovered transposon mutants were resuspended in LB broth and stored at -80°C with 15% glycerol. The final library screened for mutants with altered hemolysis was created from four batches containing a total of 177,600 mutants (i.e. the input pool). Transposon mutants with altered hemolytic activity compared to the parent strain were subcultured individually on blood agar to confirm their hemolysis phenotype, and stored individually at -80°C in LB broth containing 15% glycerol.

Overnight cultures of each transposon mutant that possessed an altered hemolytic phenotype compared to the parent S65EC strain were prepared, and a volume of 100 µl from each culture was used to make a pooled suspension of the mutant collection according to their hemolysis phenotype (i.e. the output pools). Genomic DNA was extracted from both input and output pools using the Ultraclean**^®^** Microbial DNA Isolation Kit (MO BIO). Genomic DNA from each pool was indexed and sequenced using the *Illumina* platform as previously described (11, 12). Tn*5*-specific reads were identified using the FASTX-Toolkit (v.0.0.13) and mapped to the S65EC complete genome using MAQ (v0.7.1) (13) to identify the mini-Tn*5* insertion sites.

**Targeted gene mutation and complementation.**

Mutants corresponding to selected genes identified in the TraDIS analysis were generated using λ-Red mediated homologous recombination (11, 14). Briefly, a 3-way PCR product containing the *cat* gene from pKD3 flanked by 500-bp homologous arms corresponding to the target gene was amplified and used to generate each mutant, respectively with primers listed in Table S1. Each PCR amplicon, respectively, was transformed into S65EC containing pKOBEG (harboring the λ-Red recombinase). All mutants were confirmed by Sanger sequencing. Plasmids for complementation experiments were generated by PCR amplification and cloning of each respective gene into plasmid pSU2718. Genes were amplified from S65EC using primers containing restriction enzyme recognition sites (listed in Table S1). All plasmids were confirmed by sequencing. Plasmids were transformed into the corresponding mutants; gene expression was induced with 1 mM IPTG when necessary.

**Generation of plasmids containing variant *hlyCABD* alleles**

The *hlyCABD* operon was PCR amplified from S65EC, S115EC, HVM277 and HVM2044 with primers 5462_SacI-RBS_hlyC_F and 5463_XbaI_hlyD_R (Table S1). The cloning vector pSU2718 and each *hlyCABD* amplicon and were digested with SacI and XbaI, and subsequently ligated to generate pHly^S65EC^, pHly^S115EC^, pHly^HVM277^ and pHly^HVM2044^, respectively. The plasmids were then transformed to MG1655. All recombinant plasmids were confirmed by sequencing.

**Sample preparation for western blot**

Bacterial strains were grown in 30 ml of LB with 10 mM CaCl_2_ to the late log-phage (OD_600_ = 0.9 - 1) (supplemented with antibiotics when necessary). Bacterial cells were harvested, and the cell pellets were resuspensed in 300 µl of TCU buffer (150mM NaCl, 6M urea, 20mM Tris, pH7.0) (15). The supernantants were filtered and secreted proteins were pelleted using ammonium sulfate 60% (w/v) overnight at 4°C. Precipitated proteins were pelleted by ultracentrifuge (59,000 *x g*) for one hour at 12°C, and resuspended in 300 µl of TCU buffer. Secreted proteins and the cell lysates were boiled for 10 minutes before being electrophoresed on a 12% SDS-PAGE gel and transferred to a polyvinylidene difluoride membrane. The membrane was treated with monoclonal antibody H10 against HlyA as the primary antibody (1:20,000 dilution), and subjected to alkaline phosphatase-conjugated anti-mouse IgG as the secondary antibody. Sigma Fast BCIP/NBT (5-bromo-4-chloro-3-indolylphosphate/Nitro Blue Tetrazolium) was used as the substrate in the detection process. Anti-OmpA antibody was used as a loading control.

**References**

1. Hohenhaus DM, Schaale K, Le Cao KA, Seow V, Iyer A, Fairlie DP, Sweet MJ. 2013. An mRNA atlas of G protein-coupled receptor expression during primary human monocyte/macrophage differentiation and lipopolysaccharide-mediated activation identifies targetable candidate regulators of inflammation. Immunobiology 218:1345-53.

2. Murthy AMV, Sullivan MJ, Nhu NTK, Lo AW, Phan MD, Peters KM, Boucher D, Schroder K, Beatson SA, Ulett GC, Schembri MA, Sweet MJ. 2019. Variation in hemolysin A expression between uropathogenic Escherichia coli isolates determines NLRP3-dependent vs. -independent macrophage cell death and host colonization. FASEB J doi:10.1096/fj.201802100R:fj201802100R.

3. Li H, Durbin R. 2009. Fast and accurate short read alignment with Burrows-Wheeler transform. Bioinformatics 25:1754-60.

4. Walker BJ, Abeel T, Shea T, Priest M, Abouelliel A, Sakthikumar S, Cuomo CA, Zeng Q, Wortman J, Young SK, Earl AM. 2014. Pilon: an integrated tool for comprehensive microbial variant detection and genome assembly improvement. PLoS ONE 9:e112963.

5. Seemann T. 2014. Prokka: rapid prokaryotic genome annotation. Bioinformatics 30:2068-9.

6. Zhou Y, Liang YJ, Lynch KH, Dennis JJ, Wishart DS. 2011. PHAST: A Fast Phage Search Tool. Nucleic Acids Res 39:W347-W352.

7. Altschul SF, Gish W, Miller W, Myers EW, Lipman DJ. 1990. Basic local alignment search tool. J Mol Biol 215:403-10.

8. Alikhan NF, Petty NK, Ben Zakour NL, Beatson SA. 2011. BLAST Ring Image Generator (BRIG): simple prokaryote genome comparisons. BMC Genomics 12:402.

9. Carver T, Berriman M, Tivey A, Patel C, Bohme U, Barrell BG, Parkhill J, Rajandream MA. 2008. Artemis and ACT: viewing, annotating and comparing sequences stored in a relational database. Bioinformatics 24:2672-6.

10. Sullivan MJ, Petty NK, Beatson SA. 2011. Easyfig: a genome comparison visualizer. Bioinformatics 27:1009-10.

11. Phan MD, Peters KM, Sarkar S, Lukowski SW, Allsopp LP, Gomes Moriel D, Achard ME, Totsika M, Marshall VM, Upton M, Beatson SA, Schembri MA. 2013. The serum resistome of a globally disseminated multidrug resistant uropathogenic Escherichia coli clone. PLoS Genet 9:e1003834.

12. Hancock SJ, Phan MD, Peters KM, Forde BM, Chong TM, Yin WF, Chan KG, Paterson DL, Walsh TR, Beatson SA, Schembri MA. 2017. Identification of IncA/C Plasmid Replication and Maintenance Genes and Development of a Plasmid Multilocus Sequence Typing Scheme. Antimicrob Agents Chemother 61.

13. Li H, Ruan J, Durbin R. 2008. Mapping short DNA sequencing reads and calling variants using mapping quality scores. Genome Res 18:1851-8.

14. Datsenko KA, Wanner BL. 2000. One-step inactivation of chromosomal genes in Escherichia coli K-12 using PCR products. Proc Natl Acad Sci U S A 97:6640-5.

15. Gonzalez-Carrero MI, Zabala JC, de la Cruz F, Ortiz JM. 1985. Purification of alpha-hemolysin from an overproducing E. coli strain. Mol Gen Genet 199:106-10.
