# Supplementary material for: Complex Multilevel Control of Hemolysin Production by Uropathogenic Escherichia coli
Source: mBio. 2019 Oct 1;10(5):e02248-19. doi: 10.1128/mBio.02248-19 (PMC6775461; doi:10.1128/mBio.02248-19)
Supplement: TABLE S1 [file mBio.02248-19-st001.docx]

**Table S1. Primers used in the study.**

| **Primers** | **Sequence 5' to 3'** |
| --- | --- |
| **Primer for qRT-PCR** |  |
| hlyC.F | TGGTTCATTGACTGGATTGC |
| hlyC.R | GTTTTGGGATCCACCCTG |
| hlyA.F | TTGTCAGGACGGCAGATG |
| hlyA.R | TGTGGTGCAAAGATAGTCACTC |
| 4774-gapA-F | CGTTAAAGGCGCTAACTTCG |
| 4775-gapA-R | ACGGTGGTCATCAGACCTTC |
| **Primers to identified transcriptional start site of hlyC** | |
| hlyC_GSP1 | TACCTCCGTGAAATTCTG |
| hlyC_GSP12 | AACAGGTTAGCGCATTAC |
| hlyC_GSP14 | CACATACCATCCTTGCAC |
| **Primers to generate and confirm defined mutants** | |
| 3746-Cm.3a | TCCTCCTTAGTTCCTATTCC |
| 3747-Cm.4a | GTCTTGAGCGATTGTGTAGG |
| 3748-Cm.up | CCTGAAAATCTCGACAACTC |
| 3950-Cm.dn.new | GGTTCATCATGCCGTTTGTG |
| 7113-hlyA-Fwsc | GCCAGTTCCCCATTACACAG |
| 7114-hlyA-Fwup | CTGCAATACGGGCTAACCAA |
| 7115-hlyA-Rvup | GGAATAGGAACTAAGGAGGA GGATTGCTTTGCAGACTGTAGTG |
| 7116-hlyA-Fwdn | CCTACACAATCGCTCAAGAC TCCGGTAATGCCAGTGATT |
| 7117-hlyA-Rvdn | GACGTCCATCCTCTCTCCAG |
| 7118-hlyA-Rvsc | TGCCCCTGATATAACGCCTC |
| 8342-rfaE-Fwsc | AGTACGCCGGGAGATTATGA |
| 8343-rfaE-Fwup | AAACAGAAGTGCGGGAAATG |
| 8344-rfaE-Rvup | GGAATAGGAACTAAGGAGGA ATCCAGCATCACATCACCAA |
| 8345-rfaE-Fwdn | CCTACACAATCGCTCAAGAC TTGAAGACGGTTGCTCGAC |
| 8346-rfaE-Rvdn | ATAATCCCTGGCCCAAAAAG |
| 8347-rfaE-Rvsc | GCACTCTCTTACCGCACACA |
| 8318-waaC-Fwsc | TGTACGCGTGCTCGATAAAG |
| 8319-waaC-Fwup | GAACGTCCGATGATTGGTTT |
| 8320-waaC-Rvup | GGAATAGGAACTAAGGAGGA AACGATCAGAACCCGCATC |
| 8321-waaC-Fwdn | CCTACACAATCGCTCAAGAC TCAACAGTCAAGCAGTTTTGG |
| 8322-waaC-Rvdn | CCAGCAATTAGAAATGACAGAGAA |
| 8323-waaC-Rvsc | GCCGTTCCTACACCAAAAGA |
| 8348-waaF-Fwsc | AAGCGAACTCGCAGATTGTT |
| 8349-waaF-Fwup | GACCTCGTGAAGGCCATAAA |
| 8350-waaF-Rvup | GGAATAGGAACTAAGGAGGA GCGATAGAGACTTTGCGACA |
| 8351-waaF-Fwdn | CCTACACAATCGCTCAAGAC CACCAGAGCTTGATCGACAT |
| 8352-waaF-Rvdn | ACAGGCTGGCTAACGGTTC |
| 8353-waaF-Rvsc | CCAGCATCTGTAGGCAGGTT |
| 3644-waaG-Fwsc | GTGAAGCAGACAACCATGAG |
| 3645-waaG-Fwup | TTCAAATGCTGGGACAACG |
| 3646-waaG-Rvup | GGAATAGGAACTAAGGAGGAAACGATCATAACGACGTACC |
| 3647-waaG-Fwdn | CCTACACAATCGCTCAAGACGGTGGTCTGGATGGTTGAAC |
| 3648-waaG-Rvdn | AGTGCAGCAGGAAATGACAG |
| 3649-waaG-Rvsc | CAAATCTTTATCCCGCCAAC |
| 3386-dnaJ-Fwsc | GAAAGACAAAAACAGCGGTA |
| 3387-dnaJ-Fwup | AAATCCAGAAAATGGTACGC |
| 3388-dnaJ-Rvup | GGAATAGGAACTAAGGAGGA GGAAACGCCTAAAATCTCGT |
| 3389-dnaJ-Fwdn | CCTACACAATCGCTCAAGAC TAACCTCCCCAAAAGCCTGC |
| 3390-dnaJ-Rvdn | CCTTTTTTGTGAATCCCTGG |
| 3391-dnaJ-Rvsc | CTGCTGAACGTATTTCTTTG |
| 8448-acrR-Fwup | GCTTCGATGTCGCTACCTTC |
| 8449-acrR-Rvup | ggaataggaactaaggaggaTGAGAAAAGACGTAGAGCCACA |
| 8450-acrR-Fwdn | cctacacaatcgctcaagacGCTTCGTAACCCTGCCACTA |
| 8451-acrR-Rvdn | CAGAATTTTGCGCGTTTCTT |
| 8362-acrR-Fwsc | CTTTCGCCAGATCACCTTTC |
| 8367-acrR-Rvsc | TGCGTCTGTAACGAAACCAG |
| 8596_04585_Fwup | CCTGACGACGAAACAACG |
| 8597_04585_Rvup | GGAATAGGAACTAAGGAGGA TTTTAACGGTAATGGCTGC |
| 8598_04585_Fwdn | CCTACACAATCGCTCAAGAC CTGTTATTTTAGATGGCGCTG |
| 8599_04585_Rvdn | AAGGCATTAAAAGGGAATGG |
| 8600_04585_Fwsc | CAGATCAAACTCGCGTGG |
| 8601_04585_Rvsc | TTGCACTCCATCCTTCAACAC |
| 8368-04586-Fwsc | TGTAGTGGATGGCGCATTTA |
| 8369-04586-Fwup | TGGCTTGGTGGTGCGTTA |
| 8370-04586-Rvup | GGAATAGGAACTAAGGAGGAATCTTTCACCACGTAATGGG |
| 8371-04586-Fwdn | CCTACACAATCGCTCAAGACAAAACCCCAAGCATGTTCAG |
| 8372-04586-Rvdn | CAGAGCCTGACATTGCCATA |
| 8373-04586-Rvsc | GCGCGGTAATAATTTTCAGG |
| **Primers to clone genes into pSU2718** | |
| SacI-RBS_hlyC_F | GCGGAGCTCTTAAAGAGGAGAAAGGTACCGCTTGGTTTGCTTTTTTTTACCTGC |
| XbaI_hlyD_R | GGGATCTAGATTAACGCTCATGTAAACTTTCTGTTAC |
| BamHI-RBS-rfaE_F | ATCGCGCGCAGGATCCTTAAAGAGGAGAAAGGTACCATGAAAGTAACGCTGCCAG |
| XbaI-rfaE_R | TGATGATGTCTAGATTAGCCTTTTTTATCCAGTTGGA |
| BamHI-RBS-waaF_F | ATGGCCTGGGATCCTTAAAGAGGAGAAAGGTACCGCATGAAAATACTGGTGATCG |
| HindIII-waaF_R | TCAGAACCAAGCTTCGTCAGGCTTCCTCTTGTAAC |
| BamHI-RBS-waaG_F | CATTGCAGGGATCCTTAAAGAGGAGAAAGGTACCACGACAGGTACGTCGTTATG |
| HindIII-waaG_R | ACGGCTCTAAGCTTTCAACCATCCAGACCACC |
| XbaI-RBS-waaC_F | CAACGCGCTCTAGATTAAAGAGGAGAAAGGTACCATGCGGGTTCTGATCGT |
| SalI-waaC_R | ATGAATGAGTCGACAAGGATGTTAGCATGTTTTACC |
| BamHI-RBS-dnaJ_F | CGTGCGGATCCTTAAAGAGGAGAAAGGTACCATGGCTAAGCAAGATTATTACGAG |
| XbaI-dnaJ_R | GCTTTTGTCTAGATTAGCGAGTCAGGTCGTCG |
| 4481 | AAAGGGGGATGTGCTGCAAG |
| 4482 | TCCGGCTCGTATGTTGTGTG |
